# Assessment of a support garment in parastomal bulging from a patient perspective: a qualitative study

**DOI:** 10.1080/17482631.2022.2039428

**Published:** 2022-02-17

**Authors:** Trine Borglit, Marianne Krogsgaard, Stine Zeberg Theisen, Mette Juel Rothmann

**Affiliations:** a Department of Surgery, Zealand University Hospital, Koege, Denmark; b Department of Quality, Holbaek Hospital, Holbaek, Denmark; c Steno Diabetes Center Odense, Odense University Hospital, Odense, Denmark

**Keywords:** parastomal hernia, parastomal bulging, stoma, support garment, assessment, qualitative, patient perspective

## Abstract

**Aim:**

To investigate patients’ experiences of the assessment of support garments in relation to a parastomal bulge.

**Methods:**

We conducted a qualitative study with semi-structured interviews preceded by field observations. The 11 in-dept interviews were analysed using interpretative phenomenological analysis.

**Results:**

In the assessment process patients lacked information from professionals on the advantages, disadvantages as well as criteria for choosing between garments.

Garments had to fit patients’ needs and personal preferences; being comfortable, flexible and user-friendly. The garment created new possibilities and challenges; well-assessed garments reduced symptoms while poorly assessed worsened or induced symptoms and ended up unworn. When comorbidities were not accounted for, garments were unmanageable to patients. Patients needed guidance on how to apply and use the garment. Lack of hands-on-guidance left patients confused and helpless with unworn garments. Re-assessment of a garment before it could be worn was time consuming, stressful and required patients’ physical and mental resources.

**Conclusion:**

Exploring patients’ expectations, symptoms, needs and comorbidity was vital for patients’ subsequent use and benefit of garments. Tailor-made information, hands-on-guidance and professional assistance are important in the assessment process. Interventions to support a patient centred, individual and systematic approach is warranted.

## Introduction

### Background

Despite advancements in the creation of enterostomies, parastomal bulging is a frequent long-term complication following formation of a stoma after elective and emergency abdominal surgery (Carlsson et al., 2016; Jones et al., 2018; Osborne, 2017; Sjodahl et al., 2011). A parastomal bulge is a visible or palpable bulge at the site of the stoma. The term parastomal bulge includes mainly true parastomal hernia and “pseudohernia” due to a subcutaneous prolapse, but also the infrequently reported weak abdominal wall (Rubin, 2004; Smietanski et al., 2014). Clinically, it is difficult to differentiate between types of parastomal bulging (Rubin, 2004). Further, for parastomal hernia and subcutaneous prolapse, patients symptoms are likely to be overlapping and known to impact patients’ quality of life (Kald et al., 2008; Krogsgaard et al., 2019). More than 50% of patients with a permanent colostomy develop a parastomal bulge, and in patients with an ileostomy, it accounts for more than a quarter (Antoniou et al., 2018; Glasgow & Dharmarajan, 2016; Harb, 2018; Shabbir & Britton, 2010; De Smet et al., 2020). The prevalence of parastomal bulge increases the longer the stoma is in situ (Antoniou et al., 2018). Surgical repair may be associated with high risk of mortality, morbidity and recurrence (Helgstrand et al., 2013; Kroese et al., 2018) leaving the majority of patients to live with their parastomal bulge and the related symptoms (Cowin et al., 2012; Harb, 2018; Krogsgaard et al., 2019). Stoma patients consult a stoma nurse for regular or needs-based follow-up in relation to early and late complications, including parastomal bulging (Krogsgaard et al., 2017a). In the non-operative management of parastomal bulging stoma nurses provide knowledge, advice and practical solutions to minimize symptoms and discomfort. Support garments are one of the most frequently recommended aids in stoma care for patients with a parastomal bulge (Osborne, 2017). When patients are prescribed a garment it can either be measured and fitted by a stoma nurse or a manufacturer. Typically, the garment is subsequently sent to patients by post.

Wearing a support garment can contribute to a feeling of safety, looking normal (Hubbard et al., 2019; Johnson et al., 2015) and conceal the parastomal bulge (Hubbard et al., 2019; Krogsgaard et al., 2017a). Physical symptoms such as pain (Krogsgaard et al., 2017b), a bearing-down or falling out sensation (Hubbard et al., 2019; Krogsgaard et al., 2017a) can be relieved. A garment might prevent or contain a leakage of the stoma (Hubbard et al., 2019). Some patients wear the garment to reduce the risk of developing a parastomal bulge or prevent enlargement of a bulge (Hubbard et al., 2019) although this is not supported by evidence (Krogsgaard, 2020).

A garment is an adjustable belt or pants which are either a standard manufactured product or a bespoke garment custom made for the individual. Garments are available in different lengths, depths, colours and designs and may or may not be constructed with a hole that allows the pouch to be pulled through and worn outside the garment (Hubbard et al., 2019; Readding, 2014). Individual assessment is essential to ensure the best fit of the garment so as to manage symptoms from a parastomal bulge (Hubbard et al., 2019; Readding, 2014). As a result of the difficulties healthcare professionals have experienced in supporting these patients (Osborne, 2017) stoma care nurses have expressed a need for increased knowledge and greater competencies in measuring and fitting support garments. In a survey of patients with a parastomal bulge, who ordered a garment, only 45% reported using the garment regularly (Cowin et al., 2012). Other studies described garments as uncomfortable to wear and associated with negative bodily sensations (Hubbard et al., 2019; Krogsgaard et al., 2017a). The difficulties reported by patients may reflect that prescribed garments did not fulfil patient’s needs. Exploring patients’ experiences of the assessment process might thus provide healthcare professionals with a better understanding of garment-related needs and challenges. This is important in order to offer adequate support and treatment to patients with a parastomal bulge. Hence, the aim of this study was to explore patients’ experiences of the assessment of a support garment in relation to a parastomal bulge.

## Methods

### Design

This is a qualitative study with in-depth semi-structured interviews inspired by a phenomenological and hermeneutical position. The study is reported according to the COREQ guidelines (Tong et al., 2007).

### Setting and participants

Stoma nurses at five outpatient stoma clinics in the Capital Region of Denmark recruited patients referred to nonsurgical treatment of a parastomal bulge. Patients were approached face-to-face. Participants were over the age of 18 and able to provide informed consent. Patients were excluded if they had a urostomy or a major incisional abdominal hernia in a separate incision. Participants were contacted to arrange a suitable time for an interview. All patients were purposively recruited between September 2018 and February 2019. In total, 12 patients were approached, and we obtained informed consent from 11. Five men and six women aged 47–87 years, participated in the study. Patient characteristics are presented in Table I.

**Table I.** Patient characteristics

Female/ Male (n)	6/5
**Age** (years, range)	[47–87]
**Ostomy type** (n) Colostomy Ileostomy	7 4
**Ostomy indication** (n) Benign Malignant	4 7
**Bulge size** (n) < 10 cm >10 cm	2 9
**Cohabitant/** **Married** (n) (yes/no)	5/6
**Employment status** (n) Retired Unemployed	10 1
**Daily use of a garment** (n) (yes/no)	6/5
**Standard garment/ bespoke garment** (n)	7/4

### Data collection

Data collection was carried out in January and February 2019 by one of the first authors (TBB) and third author (SZT), who both have many years of experience in clinical nursing. Both have limited experience in conducting qualitative research and received supervision from the experienced members of the research team. In-depth semi-structured interviews were chosen, as it allows insight into patients’ experiences (Kvale & Brinkmann, 2014). No relationship with the participants was established prior to the interviews. Patients were informed about the purpose of the study. The interviews took place in patients’ home. A preceding systematic literature review and a small field study conducted before study start informed the main topics in the interview guide (Kvale & Brinkmann, 2014). Main topics were: 1) Patients’ experiences in relation to the process of having a garment assessed and 2) Patients’ experiences of using a newly assessed garment in relation to symptoms from the parastomal bulge. Field notes were made after each interview in order to record contextual details about the process and participants, including non-verbal expressions (Tong et al., 2007).

All interviews were digitally recorded, transcribed verbatim and names were anonymized. In an iterative process, the content of each interview was immediately discussed between the authors, and patients in subsequent interviews were prompted to elaborate on topics that emerged in previous interview (Kvale & Brinkmann, 2014). Transcripts were not returned to patients for comment. The interviews lasted 40–70 minutes and resulted in 160 verbatim transcribed pages. After 11 interviews data were “rich” with detailed descriptions and no new knowledge was being generated (Tong et al., 2007).

### Data analysis and interpretation

The data were analysed using the six-step guide for interpretative phenomenological analysis (IPA; Smith et al., 2009) shown in Table II. Steps one to four in the IPA process were handled separately for each interview by the authors (TBB and SZT). Subsequently, the analysis was discussed jointly. The analytical process involved reading the transcripts several times, followed by open coding with focus on the descriptive, linguistic and conceptual comments, leading to superordinate themes (Smith et al., 2009). Furthermore, there was a focus on identification of convergence and divergence in the interviews. Three main themes were derived. The analysis was discussed with two senior researchers to strengthen the credibility of the results. Furthermore, credibility checks were integrated in the analysis by going back and forth between data and interpretations (Tong et al., 2007). The analysis of data was done manually.

**Table II.** An example of the 6 steps in interpretative phenomenological analysis (IPA)

Single-interview-analysis					Cross-interview-analysis	
Step 1: Reading and re-reading	Step 2: Initial noting (Left margin)	Transcription	Step 3: Emergent themes (Right margin)	Step 4: Super-ordinate themes	Step 5: Moving to the next case	Step 6: Final theme
Repeated review readings	Describes that the garment does not cover the bulge and feels uncomfortable. The garment feels like a rigid corset. The feeling of not being able to move the legs when wearing the garment. Feeling frustrated.	*The garment did not cover my bulge, it was completely hopeless. It felt very uncomfortable, you know, like a rigid corset causing pain (patient 3).*	The garment must take the bulge and physical condition into account. Challenging with an ill-assessed garment. Frustration.	The importance of an individually assessed garment. The garment has to fit properly.	Pause “clear one´s mind” before moving on to the next individual transcript.	“The support garment has to fit my needs and me”.
Repeated review readings	Describes that a garment without a hole causes leakage. The patients’ clothes had to be washed because of leakage. A garment with a hole causes no leakage. Frustrated.	*It was a garment without a hole. It did not go very well, if you can put it that way. It caused leakage … all my clothes had to be washed. You know … it was awful. Then I got this one (showing a garment with a hole), and now it is working (patient 7).*	The importance of an individually assessed garment. Hole versus no hole. Frustration.	Important to take patients individually needs into account.	Pause “clear one´s mind” before moving on to the next individual transcript.	

## Ethics

All participants received oral and written information before signing an informed consent form and could at any time withdraw their participation. The study was approved by the Danish Data Protection Agency (VD-2019-01). According to Danish legislation, the study did not need approval from the National Committee on health Research Ethics. The study was conducted in accordance with the Helsinki Declaration (World Medical Association, 2000) and the Ethical Guidelines for Nursing Research in the Nordic countries (Northern Nurses’ Federation, 2003).

## Results

In the following, we represent the main themes that emerged on basis of IPA. The themes are based on the disclosures expressed by the patients and were derived from patterns across interviews. The themes were: 1) The support garment has to fit my needs and me, 2) The assessment process requires physical and mental resources, 3) The support garment creates new possibilities and challenges in everyday life.

### Theme 1: the support garment has to fit my needs and me

Consideration of patients’ individual needs was crucial to all patients in the assessment process. Patients’ type of stoma, stoma appliance, body shape, physical condition and specific symptoms in relation to the parastomal bulge had to be accounted for. Comfortableness, flexibility, and user-friendliness was also essential: *The garment has to fit me … and since it is something you have to live with for the rest of your life, you have to be sure, that it’s something that suits your body and your needs …. and your way of living (Patient 3)*. Most patients also reflected on personal preferences such as colour, design and type of material. Visibility, noise and hygiene was important, as patients did not wish to attract attention to their parastomal bulge. Patients found it challenging to obtain a material and design comfortable enough to wear and enable symptom management: *It has to look sporty and masculine. They wanted to give me a salmon-coloured one, and then I had an emotional meltdown. It very quickly becomes disgusting, as if you were a pig, right? (Patient 4)*. When garments did not fulfil essential requirements, patients abandoned using them and referred to the garments with metaphors like “iron-armor” or “corset”.

Some patients felt that their individual needs were paramount and taken into account by professionals during this process. Others felt ill prepared and sought to educate themselves using the library or the internet. They found that by doing this and having and using expert language, they were able to have their voices heard.

The majority of patients expressed a need for more information *as to what criteria should be applied* when choosing between different types of standard garments and bespoke garments. Some patients had a garment fitted with a hole while others had been given a garment without a hole and thought this was the only type available. For some patients, wearing a garment without a hole lead to leakage: *Every time I use this garment without a hole, I have a leakage. …. Otherwise, I don’t (Patient 10)*. Others experienced being dependent on help from family to apply and remove the garment every time the pouch needed to be emptied: *I need help from my husband to apply the garment every time after toilet visits (Patient 2)*. Some patients described information from professionals as being completely absent. A patient expressed not knowing the reason why she was prescribed a garment: *I don’t know, they [the stoma nurse, ed] just told me so, they just thought it would be good for me, and then I did it (Patient 8)*. This patient later gave up using the garment.

Most patients expressed a desire to try on different types of garments with the assistance of a manufacturer or stoma nurse. Patients also wished for a trial period to test the fit and feel of different garments. Not having sufficient opportunity to test and evaluate the fitted garment in everyday life resulted in either an unworn garment or patients continuing to struggle for the garment to fit properly.

### Theme 2: the assessment process requires physical and mental resources

Following the initial assessment, the garment was dispensed immediately and was ready for use for some patients. Others had delays before use, some needing to wait for their right size to be available, or for a bespoke garment to be made or re-assessment of the garment, before it could be worn. Re-assessment was described as an exhausting, stressful and time-consuming process that required patients’ physical and mental resources. The process necessitated several visits to the manufacturer, telephone calls or deliveries by post: *I have spent a lot of time. Seven times [postal deliveries back and forth, ed] … I can’t do it anymore (Patient 6)*. Receiving the garment by post without any guidance or user instructions left patients confused: *How do I apply it? (Patient 7)*. Others received the garment and a booklet in English, which for some, was a challenge: *There was an instruction manual included, but I cannot use it, it is not in Danish … (Patient 9)*. A few patients tried to get an outpatient appointment with the stoma nurse to get “hands-on-guidance” but with no success. Lack of guidance could result in a feeling of helplessness and unworn garments. Patients requested guidance in the form of a booklet with clear descriptions, pictures and links to a video.

Patients, who were able to employ their physical and mental resources, ended up with a well-assessed and usable garment. These patients were confident that they could call the stoma nurse when in doubts: *If any problems or questions, I feel I can talk to them [the stoma nurses, ed.] about it. It is very important that I know where to go when in doubts (Patient 3)*. Conversely, patients with less knowledge, confidence and assertiveness were left without guidance and ended up with costly, unworn garments and a feeling of hopelessness: *She [the stoma nurse, ed] found out that the garment was too small and sent me a larger one. I called back and told that this one was also too small and then I got a larger one by post. I couldn’t figure out how to fit it, and I gave up (Patient 8)*. For some patients it took up to nine months to be assessed, fitted and receive a bespoke garment Patients felt inadequately prepared to decide whether, yet another reassessment of the garment was necessary. Some described that even after months of re-assessments, the garment did not fit and was left unworn. Few patients did not experience any problems and described the whole assessment process as: *It has gone very well (Patient 1*).

### Theme 3: the support garment creates new possibilities and challenges in everyday life

Patients described that the garment reduced pain, irregular or absence of stool, bearing down sensation, leakage problems, discomfort in physical activities, fear of intestinal obstruction and an uncomfortable feeling of: *The “whole thing” (guts) is falling out (Patient 6)*. Furthermore, the garment reminded patients to tense the muscles during physical activity hoping it could prevent enlargement of the parastomal bulge. In this way work, hard physical activity, hobbies, being and feeling comfortable in public and shopping with friends became possible: *I have a lot of pain from my hernia, but when I wear the garment, I can manage the pain. It means that I can go shopping and have a more social life (Patient 2)*. Moreover, everyday activities like biking, playing football and cooking became easier: *I can stand by the stove and the kitchen sink without bumping into anything all the time (Patient 1)*. Wearing the garment made it easier to create a symmetrical appearance and thus easier to find suitable clothing, along with hiding the bag especially during intimacy: *So, I can hide and stop my bag from flitting around during sex (Patient 4).*

For others new challenges arose. Using a garment that did not fit the size and shape of the parastomal bulge, their body contour, stoma or type of appliance resulted in negative bodily sensations, e.g., feeling uncomfortable tight and warm, sweaty and itching skin especially in summertime: *It is awfully hot to wear. After four hours you can’t stand it anymore, it made my skin itch (Patient 11)*. Others described difficulty breathing or complications such as leakage, wounds arising around the stoma and obstruction of the blood supply to one’s legs: *The legs “locked” when the garment was on …. I couldn’t walk and had to take breaks (Patient 3).*

Some patients stopped using poorly fitting garments. Others felt they had to use the garment for managing their parastomal bulge although symptoms from the parastomal bulge worsened. Information from stoma nurses and manufactures that the garment should be worn during physical activity and could prevent incarceration and enlargement of the parastomal bulge led to feelings of guilt for some patients if they did not wear the garment daily: *If I don’t wear it (the garment) then I have a bad conscience towards my bulge (Patient 4).*

Some patients had pre-existing health challenges and comorbidities that were not taken into account when assessing the particular garment for that individual, e.g., incontinence, psoriasis, visual impairments, breathlessness, poor balance, arthritis or learning difficulties after a stroke. *Hmmm, no we have never talked about it (other medical problems). I haven’t talked to the stoma care nurses that way …. They don’t even know that I have arthritis (Patient 2)*. In patients diagnosed with urge incontinence this manifested in difficulties making it to the restroom in time. Patients suffering from decreased muscle strength, who were prescribed a garment without a hole, were challenged as they had to remove the garment each time the appliance were changed or emptied. Consequently, some patients gave up using the garment, as it was exhausting and almost impossible to handle and put on. Others experimented on their own sewed pockets into the garment, so that they did not need the assistance from friends and family when applying the garment.

## Discussion

This study describes the experiences of patients with parastomal bulge being assessed for support garments. A major finding was patients’ essential need for an individual and personalized approach, treatment and follow-up. Our findings elucidate how taking patients’ personal preferences and individual needs into account was essential in the assessment process. Applying and using a prescribed garment requires guidance and follow-up. Importantly, wearing a well-assessed garment created new possibilities in patients’ everyday lives, whereas an ill-fitting garment created even more challenges in daily life.

Involvement, individual guidance and information were prerequisites for patient’s subsequent use and benefits from the use of the garment. Exploring patient’s personal preferences and individual needs increased patient’s likelihood of ending up with a well-assessed garment that could manage symptoms from the bulge and preserve patients’ independence in stoma care. Personalization seemed to be key in the assessment process. In person-centred care patients’ values, needs and preferences are incorporated in health-care practice (Santana et al., 2018). Hence, an important first step for the health care professional seems to be exploration of whether a garment is the right solution for the patient. Eliciting patients’ symptoms systematically is vital in order to uncover whether patients most important symptoms can in fact be modified by wearing a garment. Likewise, patients’ pre-existing medical problems and level of independence must be taken into account along with the physical morphology of the abdomen and hernia (e.g., size, shape; Readding, 2014).

In the current study, some patients did not understand the purpose of having a garment prescribed which is similar to findings in post-burn injury patients, where less than half of patients had a sound understanding of the purpose of wearing a garment (Coghlan et al., 2017). Patients who are unable to understand the purpose of a treatment are unlikely to adhere to use and benefit from it (Elwyn et al., 2012). Further, some patients were willing to endure an ill-fitting garment despite complications such as wounds and leakage. Patients had been encouraged by professionals to wear the garment to avoid enlargement of the parastomal bulge and hence felt guilty when not wearing the garment. Guilt and shame are well-known phenomena in the patient-healthcare professional interaction, and healthcare professionals should be aware of even unintended inducement of these emotions (Darby et al., 2014; Speight et al., 2012). Thus, as we lack evidence for the outcomes of wearing garments (Krogsgaard, 2020), we believe patients should be made aware of the paucity of evidence and then be assisted to decide for themselves whether wearing a garment is the right solution for them. In all, patients lacked information about types of garments as well as advantages and disadvantages of wearing a garment. Previous research has stressed that patients value information on both the positive and negative aspects of stoma-care (Capilla-Díaz et al., 2019). Appropriate, clear information is a fundamental pillar in the stoma nurse–patient relationship (Capilla-Díaz et al., 2019) and according to our findings, applying this basic principle seems to be important during the assessment process to help patients make the right decisions. This is in line with the principle of shared-decision making with patients where sharing and discussing knowledge about treatment options and preferences includes exploration of risks and benefits (Elwyn et al., 2012; The Health Foundation, 2016).

The assessment process was complex, time consuming and stressful to patients, emphasizing the multifaceted aspects involved in this intervention. This finding may reflect that nurses similarly find sizing difficult and stressful (Watkins, 2010). Further, stoma care nurses have voiced the need to improve their competencies and skills in this domain (Osborne, 2017) pointing out that sizing issues exist. Theoretical and practical training of health care professionals combined with increased experience in assessments may be actions needed to improve quality of care. According to Dreyfus’ Five-stage Model, skilfulness and becoming an expert are based on a sufficient amount of experience (Park, 2015). We believe that systematic interdisciplinary cooperation between stoma care nurses and manufacturers in the assessment process has the potential to help develop these skills for the benefit of the patients.

In line with previous findings hands-on-guidance and easy access to counselling and professional help seemed important for patients to get a garment that was easy to fit and manageable in everyday life (Coghlan et al., 2017; Readding, 2014; Strigård et al., 2015). Furthermore, patients expressed the need to test and evaluate the fit and feel of different sizes and types of fabric before choosing the final garment. This aligns strongly with the basic principles in stoma care where selection of an ostomy pouching system likewise includes a period of test and trial. Tailor-made information either verbal, written, pictures or video was requested, and necessitates development of different types of high-quality information, taking patients learning strategies into account. Identifying and incorporating patients learning strategies into patient teaching is fundamental for patients to understand and manage complex treatments regimens (Falvo, 2011; Gagné et al., 2017).

A well-assessed and fitted garment created new possibilities in everyday life; optimized patients’ functional capacity, relieved symptoms from the parastomal bulge and helped patients’ resume usual social activities. In contrast, an ill-assessed and ill-fitting garment induced new challenges and restrictions in patients’ lives and lead to discomfort, worsening or inducing of symptoms. These findings are in keeping with previous studies (Hubbard et al., 2019; Krogsgaard et al., 2017a) underlining the complexity of assessing a garment that fits the patient. Moreover, our findings highlighted patients’ considerable comorbidity and underpin that different and even opposing needs must be considered and weighed up by patients and professionals in the assessment process. Eliciting patients’ expectations and assisting patients in prioritizing different needs may support patient adherence, in accordance with the principles of shared decision-making (Elwyn et al., 2012). Importantly, addressing patients’ comorbidity is a prerequisite for enabling such decisions.

## Strengths and limitations

The qualitative approach offered insight in patients’ perspectives of the assessment process of a garment. Although a small sample size, the aim was to explore patients’ experiences in the assessment process and to provide in depth and nuanced information of the phenomenon under investigation, and not generalization. However, we cannot exclude that additional data gathering could have added new data. The stringent use of IPA as a research approach has reduced the risk of biasing the analysis in a particular direction, thus improving validity (Smith et al., 2009). To increase the reliability two authors separately performed transcription and coding. Furthermore, the methodology, analysis and findings were discussed with two senior researchers strengthening the credibility of the study. The data analysis was presented in a table to ensure transparency. Quotations were used to link to the participants’ original statements to warrant validity. The majority of patient participants were elderly reflecting the increased incidence of parastomal bulging with age. The study is representative of our patient sample and population and we believe the study findings to be transferrable to this group of patients and to settings with similar outpatient follow-up provided by stoma nurses. Transferability to other units with differing follow up and manufacturer involvement is unknown.

## Conclusion

Patient involvement, individual guidance and information were key themes in the assessment process. Eliciting patients’ symptoms, comorbidity, expectations and needs were important for patients’ subsequent use of the garment in everyday life. The assessment process was complex, time consuming and required patient resources, which underpinned the importance of healthcare professionals’ competencies. An ill-assessed and ill-fitting garment complicated patients’ care of the stoma and worsened or induced symptoms. A well-assessed garment relieved symptoms from the parastomal bulge and helped patients resume daily activities and social life. Tailor-made information on benefits and disadvantages of wearing garments, together with hands-on practical guidance was requested. Close proximity of follow-up and easy access to professional assistance should be offered patients in the assessment process.

**Table:** 

**Suggested practice interventions** Assessment of a support garment in relation to a parastomal bulge should include: Eliciting and prioritizing of patients’ expectations and needs. Assessing patients comorbidity and independence. Eliciting parastomal symptoms systematically. Assessment of stoma function (consistency and regularity of stool). Assessment of appliance used (e.g., convexity). Taking the physical appearance of the abdomen and hernia into account. Information and follow-up in the assessment process should include: Easy access to professionals. Hands-on and written guidance on how to apply and use the garment. Written guidance on expectation of garment use. Information on undesirable scenarios such as leakage, wounds around the stoma and pain. Collaboration between manufacturer and stoma nurses with defined follow-up schedule for patients.
